# Modeling methylation dynamics with simultaneous changes in CpG islands

**DOI:** 10.1186/s12859-020-3438-5

**Published:** 2020-03-18

**Authors:** Konrad Grosser, Dirk Metzler

**Affiliations:** Department of Biology, Ludwigs-Maximilians Universität München, Großhaderner Straße 2, Planegg, 82152 Germany

**Keywords:** Methylation, CpG islands, Sequence evolution, Markov-chain Monte Carlo, Reversible jump

## Abstract

**Background:**

In vertebrate genomes, CpG sites can be clustered into CpG islands, and the amount of methylation in a CpG island can change due to gene regulation processes. Thus, single regulatory events can simultaneously change the methylation states of many CpG sites within a CpG island. This should be taken into account when quantifying the amount of change in methylation, for example in form of a branch length in a phylogeny of cell types.

**Results:**

We propose a probabilistic model (the IWE-SSE model) of methylation dynamics that accounts for simultaneous methylation changes in multiple CpG sites belonging to the same CpG island. We further propose a Markov-chain Monte-Carlo (MCMC) method to fit this model to methylation data from cell type phylogenies and apply this method to available data from murine haematopoietic cells and from human cell lines. Combined with simulation studies, these analyses show that accounting for CpG island wide methylation changes has a strong effect on the inferred branch lengths and leads to a significantly better model fit for the methylation data from murine haematopoietic cells and human cell lines.

**Conclusion:**

The MCMC based parameter estimation method for the IWE-SSE model in combination with our MCMC based inference method allows to quantify the amount of methylation changes at single CpG sites as well as on entire CpG islands. Accounting for changes affecting entire islands can lead to more accurate branch length estimation in the presence of simultaneous methylation change.

## Background

Epigenetic processes of DNA-methylation and - demethylation are strongly associated with differential gene expression and are essential during phenotypic development in mammals [28]. The most frequent form of methylation is the attachment of the methyl group at the fifth carbon position on a CpG site, that is, a cytosine nucleotide followed by a guanine nucleotide [7, 26, 28].

Regions in which more than 50% of the sites are either G or C are called CpG islands if the number of CpGs is greater than 60% of the expected number of CpG sites by random order [26, 28]. These regions are typically between a few hundred and two thousand base pairs in length [28]. CpG islands are involved in the regulation of gene transcription [5]. Comparisons of methylation states have been commonly applied and proved as a fruitful avenue of analysis of cell haematopoiesis [2, 33]. Pairwise comparison between cell types in different stages of differentiation or comparison between malignant and healthy cells during cancer development have provided insight into areas of transcription [2] and enabled inference of missing methylation states.

[4] have adapted phylogenetic methods to account for the tree-shaped genealogy of cell types when analyzing methylation changes during haematopoiesis. The branch lengths of the genealogy, representing expected numbers of methylation changes per site, were inferred via likelihood maximization. A common simplifying assumption in phylogenetics is that sequence positions evolve independently of each other [20]. Analogously, [4] assume that the methylation processes at all CpG sites are, conditioned on the genealogy, stochastically independent of each other. This model assumption is violated when, for example, methylation frequencies change in an entire CpG island in the course of gene regulation [5, 28].

The CpG methylation-demethylation model of [29] is based on the roles of the different DNA methylases in methylation maintenance and de-novo methylation (see also [1]). Also [16] take into account that methylation dynamics of CpG sites depend on the methylation rate in the surrounding DNA region, as well as other factors such as chromatin marks. [22] extended the model of [29] to account for the fact that the activity of enzymes that maintain methylation or lead to de-novo methylation of CpG sites depends on the methylation status of neighboring CpG sites. Further extensions of this model have been applied by [21] and [19] to disentangle the roles of the different DNA methylases. Meyer and Lacey [24] proposed another generalization of the model of [29], in which the effect of the methylation state of a CpG sites on other CpG sites depends on the distance between the sites. Other recent attempts at modeling neighboring methylation states made use of Ising models known from statistical mechanics [17]. There sequences in genomic regions are assumed to be subject to a model based likelihood governed by few parameters, resulting in an accurate characterization of methylation frequencies [17].

The models mentioned above have been very useful for the understanding of molecular mechanisms and function of DNA methylation. For the analysis of genome-wide methylation data and phylogenetic analyses thereof, however, the size of data sets necessitates simpler models that are compatible with efficient algorithms.

Our approach is to combine a model for independent methylation state evolution in CpG sites (single-site events, SSEs) with simultaneous changes of methylation states and rates within CpG islands (island-wide events, IWEs). The purpose of IWEs in our model is to cover variations in methylation frequencies among CpG islands as well as in time, that is among different cell types or other taxa in the tree of interest. An interpretation of an IWE could be, for example, that a CpG island in the promoter region of a gene becomes widely methylated or demethylated as part of gene regulation. For computational tractability, however, we neglect functional constraints in gene regulation that could lead to correlations between methylation rates among some CpG islands. We aim to both take simultaneous methylation changes into account and to propose a model that is at the same time simple enough for inference on large datasets with data from several cell types. With this, we aim to fill the gap in the literature where models have so far either just considered evolution of single sites [4], or concerned themselves with inference on smaller scales [19, 21, 24].

Like [4] we assume that CpG sites are affected by SSEs that change the state between unmethylated, methylated and partially methylated. We assume that the equilibrium probabilities of these three states and thus also the transition rates between them depend on the CpG island to which the site belongs. Conditioned on the specific rates within a CpG island, SSEs occur independently of each other. Some sequence evolution models developed for phylogenetic analyses allow that mutation rates change at random time points, see e.g. [15]. Here, we adapt this approach to CpG methylation-demethylation dynamics. We assume that CpG islands are at certain rates affected by events – the aforementioned IWEs – that change the equilibrium probabilities for all sites in the CpG island. We further assume that these events can simultaneously change the methylation states of some CpG sites in the island such that the new equilibrium probabilities apply immediately to the sites in the affected CpG island. We refer to our model allowing for both SSEs and IWEs as IWE-SSE model.

We have implemented a reversible-jump MCMC inference scheme [11, 13, 30] to fit this model to Reduced Restricted Bisulfite Sequencing (RRBS) methylation data [2, 23]. We validated the accuracy of this scheme in a simulation study. With RRBS data procured from mouse haematopoiesis [2, 4] we demonstrate that accounting for IWEs can lead to significantly different estimations of branch lengths of cell type genealogies. We further apply the inference scheme based on the IWE-SSE model to experimental data monitoring the differentiation of human germ cells in vitro [32]. We there compare it with an inference scheme without IWEs and with the lyne software package to gain independent evidence that considering IWEs improves branch estimates.

## Methods

### Structure of our methylation-demethylation model

**Fig. 1:**
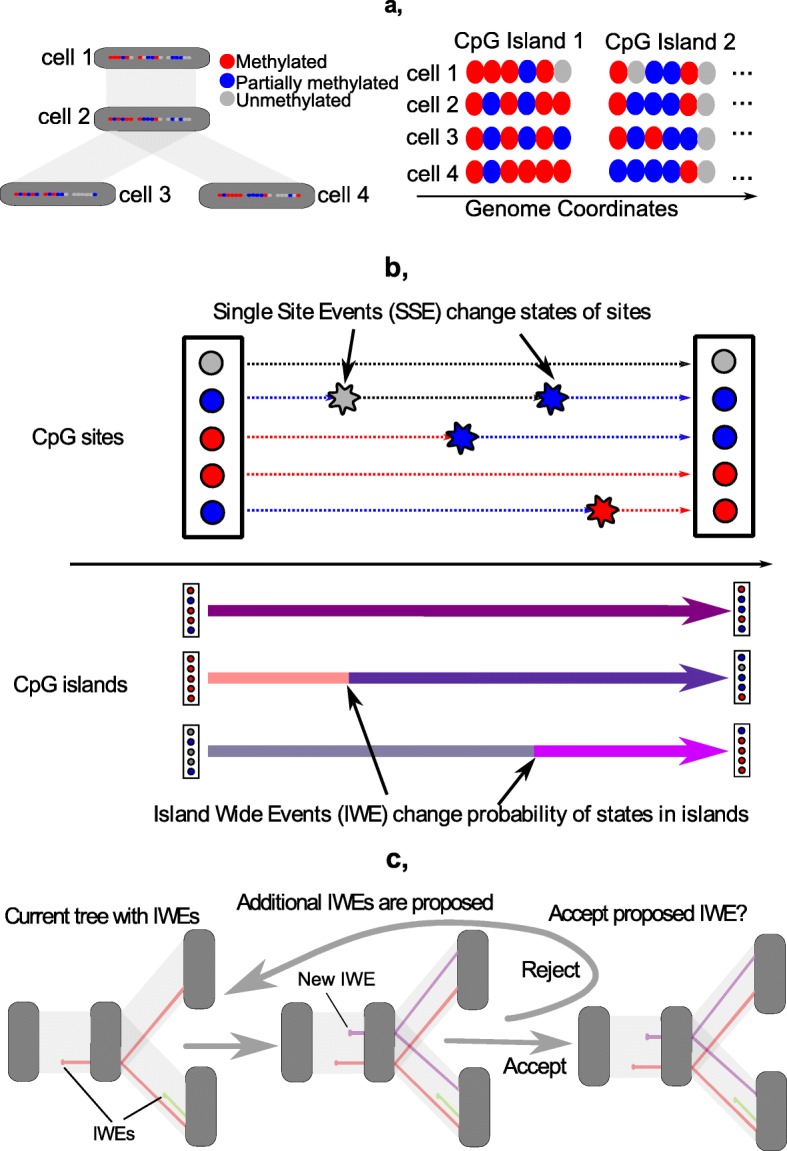
**a** We consider cells to have evolved like species in a lineage tree. Instead of DNA we consider at methylation data, where we categorize CpG sites as unmethylated, partially methylated and methylated. Sites are contained within CpG islands, which we assume to evolve independently. **b** The IWE-SSE model is two stage model that considers both events that concern individual sites (SSEs), as well as events that change the probability of methylation states (IWEs) in whole islands during evolution. **c** Location of IWEs and their corresponding probabilities are inferred by inserting proposed IWEs in a tree, and then accepting the proposal with a Metropolis Hastings acceptance probability

We take into account that several CpGs can form a CpG island, which can be affected by CpG island wide events (IWEs), in which methylation probabilities change and some of the CpG sites in the CpG island can simultaneously change their state at the same time (see Fig. 1). Different CpG islands, however, are assumed to evolve independently of each other. In addition to IWEs we allow for single-site events (SSEs), which change the methylation states of single CpG sites within CpG islands. Following [4] we distinguish three possible states {*u*,*p*,*m*} of a CpG site, denoting unmethylated, partially methylated and methylated sites. When analyzing methylation sequencing data (see “Application to the murine haematopoietic system” and “Application to in vitro cell differentiation data” sections) we classify a site as unmethylated if it is methylated in less than 10% of the reads overlapping the site, partially methylated if it is detected as methylated in 10 to 80% of the reads, and methylated if it is methylated in more than 80% of the reads.

For a branch with *h* IWEs set *t*_0_=0, let *t*_*h*+1_ be the branch length, and let $t_{1},\dots,t_{h}$ be the branch-length distances of the IWEs to the parent node with

1 $$ t_{0} \leq t_{1} \leq t_{2} \leq \cdots \leq t_{h} \leq t_{h+1}.  $$

Here we use *h*+1 as index of the branch length, since if there are *h* IWEs between a node and its parent, the resulting number of time intervals along the branch is *h*+1. For each CpG position and each open interval (*t*_*k*_,*t*_*k*+1_) there is a rate matrix *Q*_*k*_ for the transitions between the states *u*,*p*,*m*. Before we specify the details of our model for the IWE process (“Process of island-wide events (IWEs)” section) we first define our model for SSEs between given IWEs.

### Process of single-site events (SSEs)

Here we specify our model assumptions for SSEs affecting CpG sites within a CpG islands between two IWEs at time points *t*_*k*_ and *t*_*k*+1_. In the open interval (*t*_*k*_,*t*_*k*+1_), the methylation dynamics of CpGs of the same island are independent of each other and the matrix *P*_*k*_ of transition probabilities

2 $$ P_{k;i,j}=\Pr(X_{t_{k+1}}=j|X_{t_{k}}=i)  $$

between the methylation states $X_{t_{k}}$ and $X_{t_{k+1}}$ of a CpG at time points *t*_*k*_ and *t*_*k*+1_ can be calculated with the matrix exponential

3 $$ P_{k}=\exp(Q_{k} \cdot (t_{k+1} -t_{k})).  $$

In analogy to the F81 sequence evolution model [9] we focus here on rate matrices *Q*_*k*_ that can be expressed as

4 $$ Q_{k} = R\cdot \begin{bmatrix} -\pi_{p}-\pi_{m} & \pi_{p} & \pi_{m} \\ \pi_{u} & -\pi_{u}-\pi_{m} & \pi_{m} \\ \pi_{u} & \pi_{p} & -\pi_{u}-\pi_{p} \end{bmatrix},  $$

where *π*_*u*_+*π*_*p*_+*π*_*m*_=1, and each CpG has its own random rate factor *R*∈R_≥0_. Starting from any state *u*,*p* or *m* the form of *Q*_*k*_ implies that in the case of an event the state in the site becomes *u*,*p* or *m* with probabilities *π*_*u*_,*π*_*p*_, and *π*_*m*_, respectively. Note that this does not exclude the possibility that the state after the event is the same as before at the affected site. Since the three probabilities sum up to 1, the rate factor *R* directly gives the expected number of events per time unit.

The probabilities (*π*_*u*_,*π*_*p*_,*π*_*m*_) form the equilibrium distribution of *Q*_*k*_. Further, for fixed *R* the transition probabilities (*P*_*k*_)_*i*,*j*_ fulfill

5 $$ (P_{k})_{i,j} = \left(1-e^{R\cdot(t_{k}-t_{k+1})}\right)\cdot\pi_{j},  $$

if *j*≠*i*, and

6 $$ (P_{k})_{i,j} = \pi_{j}+(1-\pi_{j})\cdot e^{R\cdot(t_{k}-t_{k+1})}  $$

otherwise. We assume that in the root of the genealogy each CpG island samples the equilibrium probability triple (*π*_*u*_,*π*_*p*_,*π*_*m*_) from a uniform distribution (that is Dirichlet(1,1,1)) independently of all other CpG islands. Like in F81 and related models, the time scaling in our models can be interpreted as follows. At each CpG site with the respective rate *R*, events occur that let the CpG sample a new state *u*, *p* or *m* according to the probabilities (*π*_*u*_,*π*_*p*_,*π*_*m*_). We refer to these events as SSEs.

### Process of island-wide events (IWEs)

In each IWE a new triple of equilibrium methylation frequencies (*πu*′,*πp*′,*πm*′) is sampled from a uniform distribution, and *Q*_*t*_ is updated accordingly for time points *t* after the IWE. Furthermore, we allow that CpG sites of an island are methylated or demethylated simultaneously in an IWE in a way such that the expected frequencies of the states *u*, *p* and *m* match the new equilibrium distribution (*πu*′,*πp*′,*πm*′) right after the IWE. To specify the transition probability matrix *M*_*k*_ in an IWE at a time point *t*_*k*_, we distinguish two cases. In the first case one of the new expected frequencies is larger and the other two are smaller after the IWE. If, without loss of generality, *πu*′>*π*_*u*_, *πp*′<*π*_*p*_ and *πm*′<*π*_*m*_, then the transition matrix is

$$M_{k} = \begin{bmatrix} 1 & 0 & 0 \\ \frac{\pi_{p} - \pi'_{p}}{\pi_{p}} & \frac{\pi'_{p}}{\pi_{p}} & 0 \\ \frac{\pi_{m} - \pi'_{m}}{\pi_{m}} & 0 & \frac{\pi '_{m}}{\pi_{m}} \end{bmatrix}. $$

In the other case, one of the new expected frequencies is smaller and both others are larger. If, again w.l.o.g., *πu*′<*π*_*u*_, *πp*′>*π*_*p*_ and *πm*′>*π*_*m*_, the matrix of transition probabilities is

$$M_{k} = \begin{bmatrix} \frac{\pi '_{u}}{\pi_{u}} & \frac{\pi'_{p} - \pi_{p}}{\pi_{u}} &\frac{\pi'_{m} - \pi_{m}}{\pi_{u}} \\ 0 & 1 & 0 \\ 0 & 0 & 1 \end{bmatrix}. $$

Note that (*π*_*u*_,*π*_*p*_,*π*_*m*_)·*M*_*k*_=(*πu*′,*πp*′,*πm*′) holds in both cases. For given IWEs at time points $t_{1},\dots,t_{h}$ between time points *t*_0_ and *t*_*h*+1_, the transition matrix between the states {*u*,*p*,*m*} at time *t*_0_ and the states at time *t*_*h*+1_ is

7 $$ P_{0}\cdot\prod_{k=1}^{h}M_{k} P_{k}.  $$

### Branch length in the iWE-SSE model

For *R* in equation 4 we assume an “invariant+gamma” model [10, 34]. That is, *R* is 0 with probability *r*, and with probability 1−*r* the value of *R* comes from a discretized gamma distribution with 3 categories, expectation value 1 and a shape parameter *α*. The probability to be in each respective rate category, conditional on not being an invariant site, is 1/3.

Note that an expected fraction of

8 $$ \pi_{u}^{2}+\pi_{p}^{2}+\pi_{m}^{2}\ge1/3  $$

of the SSEs will not change the current state of the CpG, due to the probability of an SSE being in states *u*,*p* and *m* respectively being *π*_*u*_,*π*_*p*_and *π*_*m*_, and the probability of a switch to this state happening being again *π*_*u*_,*π*_*p*_and *π*_*m*_. (The lower bound of 1/3 follows considering that *π*_*u*_+*π*_*p*_+*π*_*m*_=1 constitutes a plane in 3D space whose closest distance to the origin is 3^−1/2^ and $\pi _{u}^{2}+\pi _{p}^{2}+\pi _{m}^{2}$ is the square of the euclidean distance between (*π*_*u*_,*π*_*p*_,*π*_*m*_) and the origin.)

We assume $\mathbb {E}R=1$, which implies that our time unit is the expected number of SSEs per CpG (not conditioned on *R* but averaged over the possible values of *R*). In the following, branch lengths $B:=(l_{1}, l_{2}, \dots, l_{k})$ will refer to this time scaling.

We assume that IWEs occur independently at each CpG island at rate *μ* and change the parameters values *π*_*u*_, *π*_*p*_ and *π*_*m*_ on the CpG island. For a branch of length *l* we obtain an expected number of *l* SSEs per site and of *μ*·*l* IWEs per CpG island. This implies that the branch length *l* can also be expressed as

9 $$ l=\frac{E[S+W]}{n\cdot\mu + \sum_{i=1}^{n} n_{i}},  $$

where *n* is the number CpG islands, *n*_*i*_ is the number of CpG sites on CpG island *i* with 1≤*i*≤*n* and the random variables *S* and *W* are the numbers of SSEs and IWEs on a branch of length *l*. Note here that *S* counts all SSEs, including those that do not change the methylation state of the site.

### Likelihood calculations

We summarize the global model parameters as *θ*:=(*r*,*α*,*μ*). As we assume that CpG islands evolve independently of each other, we obtain ${\Pr }_{\theta,B}(D)=\prod _{i}{\Pr }_{\theta,B}(D_{i})$, where *D*_*i*_ is the data from CpG island *i* and *B* is the vector of branch lengths of the tree. For CpG island *i* let *W*_*i*_ be the configuration of IWEs and the mutation model parameters *π*_*u*_,*π*_*p*_,*π*_*m*_ around them. That is, *W*_*i*_ can be written as

10 $$ W_{i}=(t_{1}, \pi_{u1},\pi_{p1},\pi_{m1}, \dots, t_{n_{i}}, \pi_{un_{i}},\pi_{pn_{i}},\pi_{mn_{i}}),  $$

where the *t*_*k*_ parameter refers to the position of an IWE and *π*_*uk*_,*π*_*pk*_,*π*_*mk*_ refer to its associated equilibrium frequencies. Here *n*_*i*_ constitutes the total number of IWEs that happen in the tree in island *i*. Conditioned on the configuration *W*_*i*_, the CpGs within the island become independent and we obtain ${\Pr }_{\theta,B}(D_{i}|W_{i})=\prod _{j}{\Pr }_{\theta,B}(D_{ij}|W_{i})$, where *D*_*ij*_ is the data from the *j*-th CpG in CpG island *i*.

11 $$ \begin{aligned} {\Pr}_{\theta,B}(D_{ij}|W_{i})=\qquad\qquad\qquad\\ \sum_{x} {\Pr}_{\theta,B}(D_{ij}|W_{i},R_{ij}=x) \cdot {\Pr}_{\theta,B} (R_{ij}=x) \end{aligned}  $$

where *x* is iterated over the four possible values of the rate factor *R*_*ij*_ for the CpG position. Pr_*θ*,*B*_(*R*_*ij*_=*x*) is *r* for *x*=0 and (1−*r*)/3 for the three values of *x* that are possible according to the discretized gamma distribution. To calculate Pr*θ*,*B*(*D*_*ij*_|*W*_*i*_,*R*_*ij*_=*x*) we used a recursive scheme derived from Felsenstein’s pruning algorithm [8, 10]. For this, let $D_{ij}^{(b)}$ be the part of *D*_*ij*_ that stems from the descendants of branch *b*. Let there be *h* IWEs on branch b affecting island i, with time intervals indexed by *k* between 1 to *h*+1. For an island indexed *i* and a CpG site indexed *j*, any branch *b*, state *y*∈{*u*,*p*,*m*} and *k*≥1 we now define the partial likelihood *ω*_*k*,*b*_(*y*)

12 $$ \omega_{k,b}(y):= {\Pr}_{\theta,B}(D_{ij}^{(b)} \vert W_{i}, R_{ij}, y),  $$

where $D_{ij}^{(b)}$ is the partial data, and *y* denotes the state that CpG site *j* is in just *before* IWE *k* (or the child node of *b* if *k*=*h*+1, where *h* is the number of IWEs on *b* affecting island *i*). For *k*≥0 let $\vec \omega _{k}$ be the column vector (*ω*_*k*,*b*_(*u*),*ω*_*k*,*b*_(*p*),*ω*_*k*,*b*_(*m*))^*T*^. Let $\vec \omega _{0}$ be defined accordingly, but given that the state of CpG *i* in the parent node of the branch *b* is *y*. With the transition probability matrices *P*_*k*_ and *M*_*k*_ as defined in “Process of single-site events (SSEs)” and “Process of island-wide events (IWEs)” sections we obtain

13 $$ \vec\omega_{0}= P_{0}\cdot\left(\prod_{j=1}^{k-1}M_{j}\cdot P_{j}\right) \cdot\vec\omega_{k}  $$

for any $k\in \{1,\dots,h+1\}$. The case *k*=*h*+1 is sufficient for likelihood calculations, but the formula is also used for other values of *k* for updating likelihoods when *M*_*k*−1_ and *P*_*k*−1_ are changed in an MCMC step, see online appendix section B.1.

If the child node of *b* is a tip (an external node that is not the root) of the genealogy, we obtain *ω*_*h*+1,*b*_(*y*)=1 if *y*∈{*u*,*p*,*m*} is the state of the *j*th CpG site at the child node, and otherwise *ω*_*h*+1,*b*_(*y*)=0. If *b* ends in a node with two daughter branches *b*^′^ and *b*^″^, we obtain

14 $$ \omega_{h+1,b}(y)=\omega_{0,b'}(y)\cdot\omega_{0,b^{\prime\prime}}(y)  $$

for all *y*∈{*u*,*p*,*m*}. In our application examples below, all methylation states are known not only for the tips of the tree, but also for the internal nodes and the root. In this case equation (14) holds only if *y* is the state of the *j*th CpG site at *b*’s child node, and otherwise *ω*_*h*+1,*b*_(*y*)=0. For the branch *r* that starts in the root we apply Pr_*θ*,*B*_(*D*_*ij*_|*W*_*i*_,*R*_*ij*_=*x*)=*π*_*z*,*r*_·*ω*_0,*r*_(*z*), where *z* is the state of the CpG in the root node and *π*_*z*,*r*_ is its probability according to the equilibrium distribution in the root.

### MCMC implementation

To approximate Pr*θ*,*B*(*D*_*i*_) we have to average the conditional probabilities Pr*θ*,*B*(*D*_*i*_|*W*_*i*_) over possible configurations of *W*_*i*_. For this we apply a Metropolis-Hastings MCMC method [14, 30]. Given the current configuration of *W*_*i*_ in the MCMC procedure, the proposed *Wi*′ for the next step can either lack one of the IWEs in *W*_*i*_ or have an additional IWE on some branch (see Fig. 1). Let *l* be the length of a branch *b*. As the IWE locations according to *W*_*i*_ are a priori a Poisson point process with intensity *μ*, the prior probability that *W*_*i*_ includes *n* IWEs on branch *b* is Pois_*μ*·*l*_(*n*)=(*μl*)^*n*^*e*^−*μl*^/*n*!. When the proposed *Wi*′ differs from the current *W*_*i*_ by an additional IWE on branch *b* and *n* is the current number of IWEs on this branch, the Metropolis-Hastings acceptance probability is the minimum of 1 and

15 $$ \frac{{\Pr}_{\theta,B}(D_{i}|W_{i}')\cdot\text{Pois}_{\mu l}(n+1)}{ {\Pr}_{\theta,B}(D_{i}|W_{i})\cdot\text{Pois}_{\mu l}(n)} =\frac{\Pr_{\theta,B}(D_{i}|W_{i}')}{ \Pr_{\theta,B}(D_{i}|W_{i})}\cdot \frac{\mu\cdot l}{n+1}   $$

(see online appendix B.1). If, conversely, *Wi*′ with *n*+1 IWEs on *b* is the current state, and *W*_*i*_ with one IWE less on *b* is proposed, the acceptance probability is the minimum of 1 and the inverse of any side of Eq. 15.

For the branch lengths we apply Metropolis-Hastings acceptance steps on the log scale. If *ℓ*= log(*l*) is the (natural) logarithm of the current length of a branch, the proposed *ℓ*^′^= log(*l*^′^) is drawn from a Gaussian mixture proposal distribution with density *g*_*ℓ*_(*ℓ*^′^) that is centered around *ℓ*. The proposal distribution is symmetric, that is $\phantom {\dot {i}\!}g_{\ell }(\ell ')=g_{\ell '}(\ell)$. The prior distribution of *ℓ* is a normal distribution. Let *p*(*ℓ*) denote its density (see online appendix A). When a new log length *ℓ*^′^ is proposed for a branch of log length *ℓ*, we obtain the acceptance probability

16 $$ \alpha(\ell',\ell)= \min \left\{ 1, \frac{L_{D}(l')}{L_{D}(l)} \frac{l'^{n}}{l^{n}}\cdot e^{-\mu N (l'-l)}\cdot \frac{ p(\ell')}{p(\ell)}\right\},   $$

where *n* is the current number of IWEs on the branch, *μ* is the rate of IWEs, *N* is the number of CpG islands, *L*_*D*_(*l*^′^) and *L*_*D*_(*l*) are the conditional probabilities of the data given the proposed and the current trees.

In further Metropolis-Hastings steps, the methylation state frequencies (*π*_*u*_,*π*_*p*_,*π*_*m*_) for any CpG island can be updated. Further information about priors and proposal densities can be found in online appendix A.

### Null model without iWEs

We test our model against a null model without IWEs. In the null model we still assume that each island has distinct equilibrium frequencies, which are sampled at the root from a Dirichlet(1,1,1) distribution and do not change during sequence evolution. When new branch lengths are sampled, the acceptance probability (16) in this model simplifies to

17 $$ \min \left\{ 1, \frac{L_{D}(l')}{L_{D}(l)} \cdot \frac{p (l')}{p(l)}\right\}.   $$

The parameters of the null model are the logarithms of branch lengths, the logarithm of the shape parameter of the gamma distribution of site specific rate factors, the fraction of invariant sites, and for each CpG island the equilibrium probabilities at the root states.

### Application to the murine haematopoietic system

**Fig. 2:**
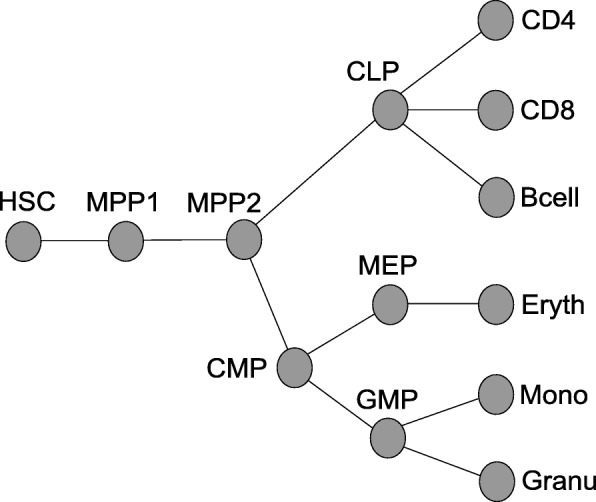
Genealogy of haematopoietic cell stages [2, 4]

**Fig. 3:**
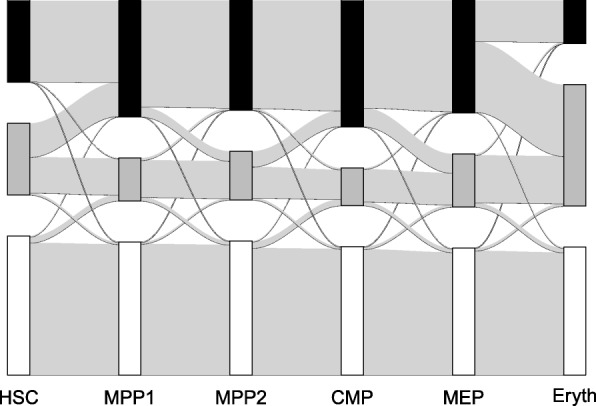
Change of Island Methylation States. States of islands are categorized as methylated (black, top) if more than 50% of sites are in state *m* and as unmenthylated (white, bottom) if more than 50% of sites are in state *u*. Otherwise, islands are categorized as partially methylated (grey, middle). Vertical rectangles are proportional in size to the respective number of islands in each state for each cell type. Light grey transitions have a width proportional to the relative amount islands that transition between the states indicated by the rectangles

We tested our approach with methylation data that were gained by [2] with RRBS from murine cells at various stages of haematopoiesis (Fig. 2). The data overlap most murine CpG islands and consist of reads that are 36 base pairs long. To associate information of reads with CpG islands we used the mmp9 mapping of CpG islands from the USCL genome browser [18]. We sampled 2000 CpG islands at random, 1970 of which contained reads overlapping CpGs within the island. CpGs were categorized as unmethylated (*u*), partially methylated (*p*) or methylated (*m*) if less than 0.1, between 0.1 and 0.8, or more than 0.8 of the reads were detected as methylated. For Fig. 3 we categorized whole CpG islands as unmethylated if more than 50% of its CpG sites were in state *u*, or as methylated if more than 50% of its CpG sites were *m*. All other CpG islands were classified as partially methylated.

### Application to in vitro cell differentiation data

In [32] human epiblast-like cells (hEpiLCs) were differentiated to primordial germ cells (hPGCs) over a twelve day period. Data about methylation levels on days 1, 2, 3 and 4 were present for hEpiLCs, as well as data for hPGCs on days 4, 5, 8 and 12. Reads were assigned to islands using mmp9 mapping and the hg37 mapping from the USCL genome browser. We randomly sampled 200 CpG islands and categorized CpG sites as explained in “Application to the murine haematopoietic system” section. We then inferred parameters based on the SSE model, based on the IWE-SSE model and with lyne as well. To evaluate how well the branch lengths inferred with each of the models reflected the temporal distance between the samples, we fitted a linear regression model without intercept between estimated branch lengths and sampling time differences (using the lm command in R [25]) and computed the fraction of explained variance (adjusted *R*^2^) as indicator of model fit. We adjusted ground truth time differences for the fact that in vivo the differentiation of hPGCs takes up to 10 days instead of 4 as it did in the induced transition cells in the in vitro environment [32].

### Application to single cell methylation data

To evaluate whether the IWE-SSE inference program behaves notably different on single cell data as opposed to bulk data, we used a dataset procured by [35].

Therein human zygotes and cells at the two cell stage where sequenced and treated with bisulfite to produce single cell data about their methylation states. Since the cell samples were not taken from the same individual, we decided to pool the zygotes into an ancestral mean population and to then infer branch lengths between this mean population and the single cells in the 2 cell stadium. To pool the zygote data we calculated the mean methylation of each site across samples and then categorized this mean into unmethylated, partially methylated or methylated. For the cells in the 2 cell stadium we categorized sites as either methylated or unmethylated depending on their methylation state. We used 100 randomly chosen CpG islands.

### Additional methods to estimate branch length

To evaluate the performance of our inference method, we compared it to two alternative approaches to estimate branch lengths. The first alternative was to use the Hamming distance which is often applied in hierarchical clustering. The second approach we took was to compute the mean methylation of the islands in each sequence. We then computed the euclidean distance between the vectors of mean values.

### Simulation study

To assess the accuracy of our MCMC implementation, we simulated 150 data sets, each consisting of 100 islands. The numbers of CpG sites in the islands were chosen randomly from a uniform distribution between 10 and 400. At the start of each simulation we sampled the logarithm of branch lengths, the logarithm of the shape parameter *α*, the invariant probability, and the IWE rate *μ* from their priors (online appendix A). For the root node we sampled equilibrium frequencies from a Dirichlet(1,1,1) distribution. Then we sampled IWEs uniformly positioned along branches, where the number of sampled IWEs on a branch was Poisson distributed with mean *μNl*, where *N* is the number of CpG islands and *l* is the branch length. The equilibrium frequencies associated with an IWE were sampled from a Dirichlet(1,1,1) distribution. We generated the sequence at the root node by drawing each state in each island from the equilibrium frequency at the root node in this island. Sequences in the other nodes were generated iteratively going from the root to the tips of the cell lineage tree using transition probabilities between states as detailed in Process of island-wide events (IWEs).

We then used our inference method on the generated sequences to find posterior distributions of the simulated data sets with known ground truths sampled from priors. Here the MCMC runs were started from the means of the priors for all parameters other than the number of IWEs, where we started without IWEs to avoid long convergence times in the case of many misplaced IWEs in the initial configuration. We used a burn-in of 10^5^ Metropolis Hasting steps. In addition to our full model we also fitted a null model without IWEs to the data.

### Test for cpG-island-wide events (IWEs)

To test the relevance of IWEs for the data of [2], we simulated 150 data sets according to this null model using 1970 islands with the same number of CpG sites as in the restricted data set we used for initial inference. These simulations were conducted with the same procedure as in the simulation study, with the starting parameters being sampled from the posterior distribution of the null model and the IWE rate being restricted to 0. We then fitted the full model with IWEs to these simulated sequences and estimated posterior number of IWEs inferred in the adapted model.

### Comparison with the r software package lyne

We conducted further simulations to compare the results of our IWE-SSE inference to results obtained with the R package lyne, which is associated to [4] and is available from the Kostka lab website^1^. We simulated branch lengths for the simplest tree that is accepted as input by lyne. This directed tree consists of a root whose sole offspring has two offspring nodes (tips). The length of the branch adjacent to the root was drawn from a log-normal distribution with mean exp(−2) and *σ*=2. Another log-normally distributed value was simulated and used the (equal) lengths of the branches adjacent to the tips, using the same distribution. The other model parameters were sampled from their priors (online appendix A). We performed 150 simulations according to the lyne model and 150 simulations following the IWE-SSE model. For the Capra-Kostka model simulations we assumed 10,000 CpG sites, which were for the purposes of IWE-SSE inference randomly sampled into islands of uniformly distributed random sizes between 10 and 200 CpG sites (but the island structure had no influence on methylation states). The IWE-SSE model simulations used 100 islands with sizes between 10 and 200 CpG sites. In silico data produced in this way was analyzed with both Kostka’s lyne package and our implementation of the IWE-SSE inference method.

## Results

### Application to methylation data from haematopoietic cells

**Fig. 4:**
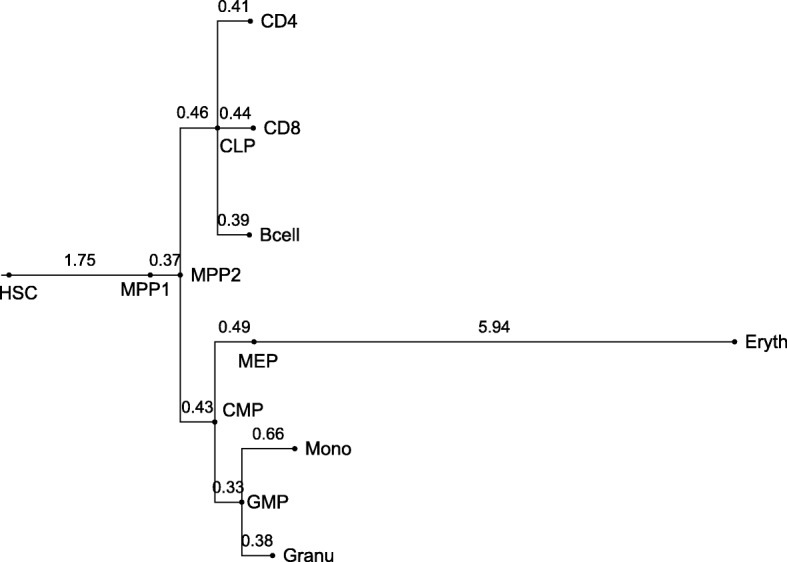
Estimates without IWEs. Tree resulting from estimates without modeling IWEs. The logarithmic branch lengths are the means of the MCMC samples after a burn in phase of 10^6^ steps

**Fig. 5:**
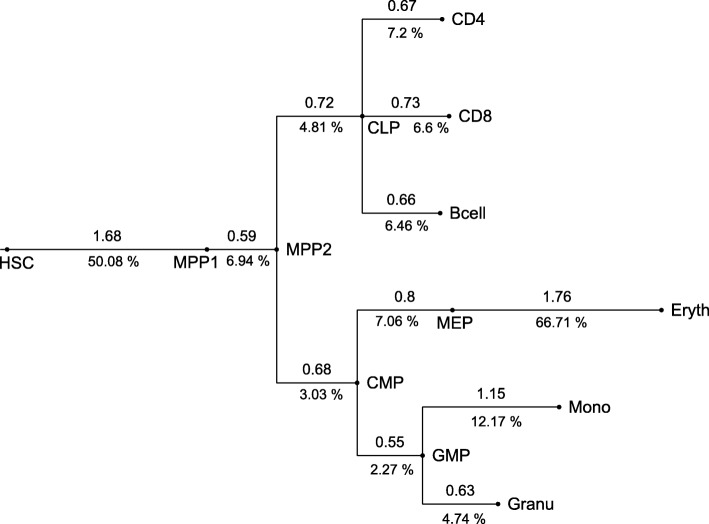
Estimates IWE-SSE model. Tree resulting from estimates modeling IWEs. The logarithmic branch lengths (above branches) and percentages of CpG islands affected by IWEs (below branches) are the means of the MCMC samples after a burn in phase of 10^6^ steps

We applied our method to 1970 randomly chosen CpG islands from the methylation data of murine haematopoietic cells [2, 4]. Using first the null model without IWEs (*μ*=0), we obtained a very long branch between MEP and Eryth indicating many changes in methylation of single CpGs (Fig. 4). Note that branch lengths are proportional to the expected number of SSEs or, in other words, to the product of cell divisions and SSE rate per cell division. We generated data following this fitted null model in 150 simulations and estimated parameters according to the model with IWEs for the null model simulations. Estimated total numbers of IWEs never exceeded 30 in any of these inferences and the inferred percentage of islands carrying an IWE along an edge was a most 0.07%. When we analyzed the data set of [2] with the IWE model (*μ*≥0), the minimum number of IWEs after the burn-in period of 10^6^ Metropolis-Hasting steps was 3488, and we inferred high levels of enrichment of IWEs on all branches (Fig. 5).

With the null model we estimated branch lengths similar to estimated lengths in the literature on these branches, e.g. between MEP and Eryth 4.56 units by Capra and Kostka, compared to a distribution mean of 5.94 SSE units with our null model. Here, an SSE unit refers to the expected number of SSEs per CpG, whereas Capra an Kostka’s unit refers to the expected number of methylation state changes per CpG. As at least a third of the SSEs do not change the state of a CpG, and 5.94·2/3=3.96, our estimation of the length of the MEP-Eryth branch is smaller than that of Capra and Kostka, but the values are not directly comparable because the model of Capra and Kostka is more general than our null model without IWEs.

When we allowed for IWE events, we found considerably less variation among the inferred branch lengths (Fig. 5). Regarding the number of IWEs, the formation of the first multipotent progenitor cells from haematopoietic stem cells and the formation of erythrocytes showed an increased frequency of such events, explaining the methylation changes between MEP and Eryth by simultaneous methylation changes in IWEs rather than by many independent single-site events.

#### Evidence that iWE rate vary among branches

**Fig. 6:**
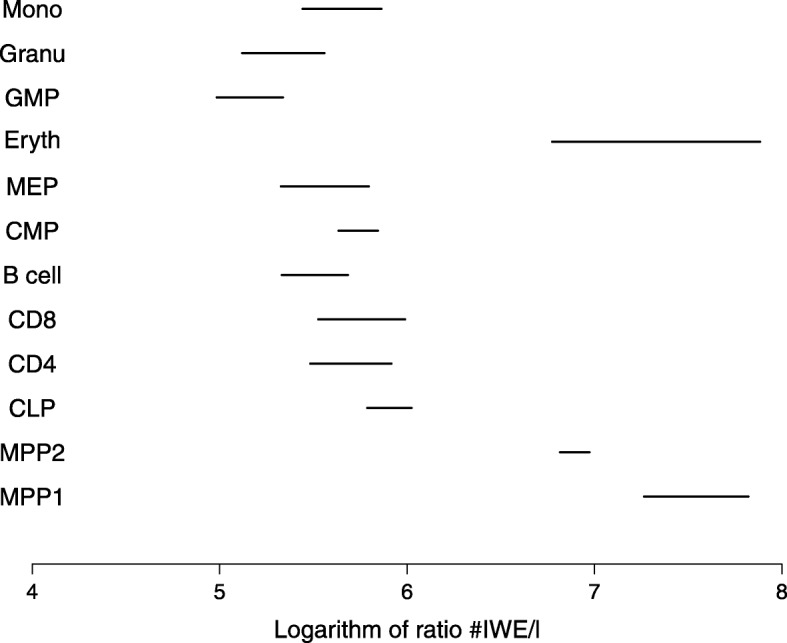
Multiple testing corrected 95% intervals of the ratio of estimated number of events to the estimated branch length

In the tree that we inferred with the IWE model (Fig. 5), the estimated numbers of IWEs vary among the branches more than the branch lengths. Indeed, credibility intervals of the log-transformed numbers of IWEs per branch length unit (Fig. 6) suggest that the IWE rate is substantially increased during the transitions from HSC to MPP1, from MPP1 to MPP2 and from MEP to Eryth. This is indicative of pronounced regularly activity along these transitions in particular (see also Fig. 3).

### Application to in vitro cell differentiation data

**Fig. 7:**
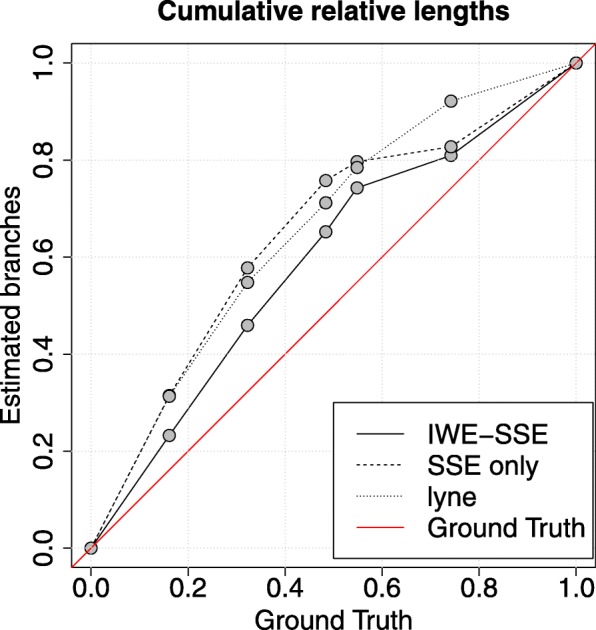
Cumulative relative length of estimates compared to known ground truth during the transition of hEpiLCs on day 1 to hPGCs on day 12. Branches in the hEpiLC stage are underestimated by all three inference tools, even though they were adjusted to match in vivo conditions

We applied our method to 200 randomly chosen CpG islands from the methylation data of human cell cultures [32]. Here we found that branch lengths in the EpiLCs were overestimated by all three inference approaches, whether we were using adjusted or experimental time spans for this stage (Fig. 7). The *R*^2^ values (that is, fractions of explained variance) we found with branch lengths were 0.40, 0.24 and 0.18 for the IWE-SSE, SSE only and lyne inference respectively. Fits improved, with the IWE-SSE model again giving the best results, after adjusting for the induced differentiation taking less time than the in vivo differentiation by multiplying the EpiLC branch lengths by 2.5 (since differentiation takes 10 days under natural conditions instead of 4 days). The *R*^2^ values we found with adjusted branch lengths were 0.80, 0.67 and 0.57 for the IWE-SSE, SSE only and lyne inference respectively. Nonetheless there was still considerable deviation from linearity, with the lengths in the EpiLC stages being overestimated for all inferences. We further applied the nonparametric Hamming distance and the euclidean mean difference for this analysis as described in “Additional methods to estimate branch length” section (see online appendix Fig. 7). Using the same linear comparison approach to a linear model without intercept, the adjusted *R*^2^ were 0.55 and 0.68 for Hamming distance and euclidean mean distance respectively.

### Application to single cell embryonic data

we applied our method to single cell data procured by [35]. We found that the estimated branch lengths were all very similar in length, as expected from their phylogenetic position (online appendix Fig. 8).

### Simulation experiments

**Fig. 8:**
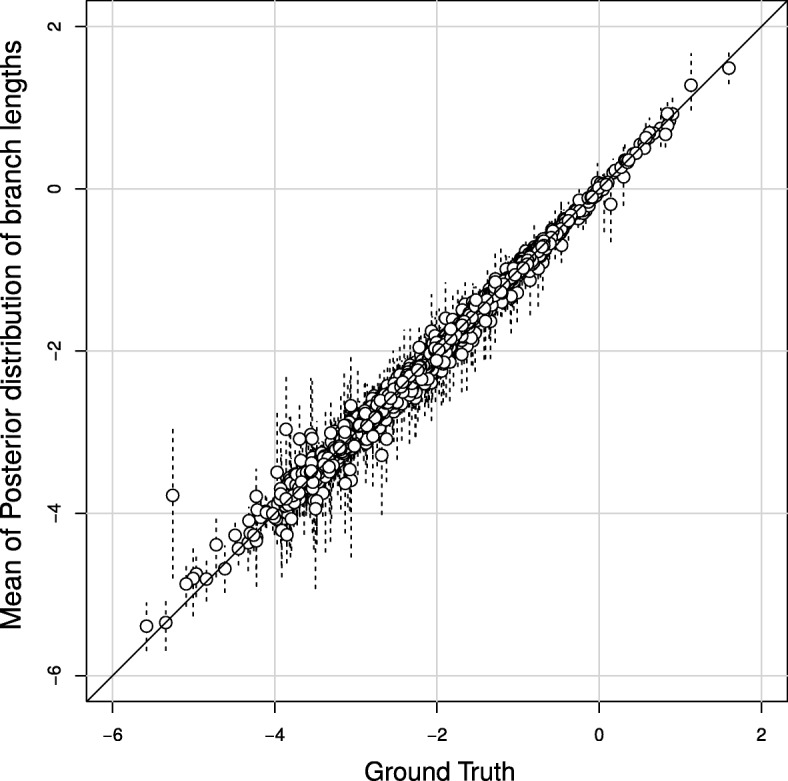
Comparison between estimated logarithms of branch lengths and target values in simulation study. Dotted lines indicate 95% credibility intervals

To validate the accuracy of our inference method, we simulated 150 data sets with parameters values drawn from the prior distributions (see MCMC implementation and online appendix A). Each of the simulated data sets contained 100 CpG islands with sizes varying uniformly between 10 and 400. For each of the simulated data sets we inferred the posterior distribution of the parameters. In Fig. 8 we compare the MCMC-sampled parameter values and credibility intervals to the actual parameter values underlying the simulations. To validate our implementation we computed the 95% credibility intervals and verified that the ground truth was within these intervals in approximately 95% of the cases. This was done for individual branch lengths, all branch lengths, the rate of IWEs and the shape parameter of rate heterogeneity. Indeed, credibility intervals overlapped the true branch length in 93 to 98% of the cases. Overall, 95% of the credibility intervals contained the true value. True values were in the credibility intervals in 96% of the cases for IWE rates and in 94% for the shape parameter.

### Comparison with lyne

**Fig. 9:**
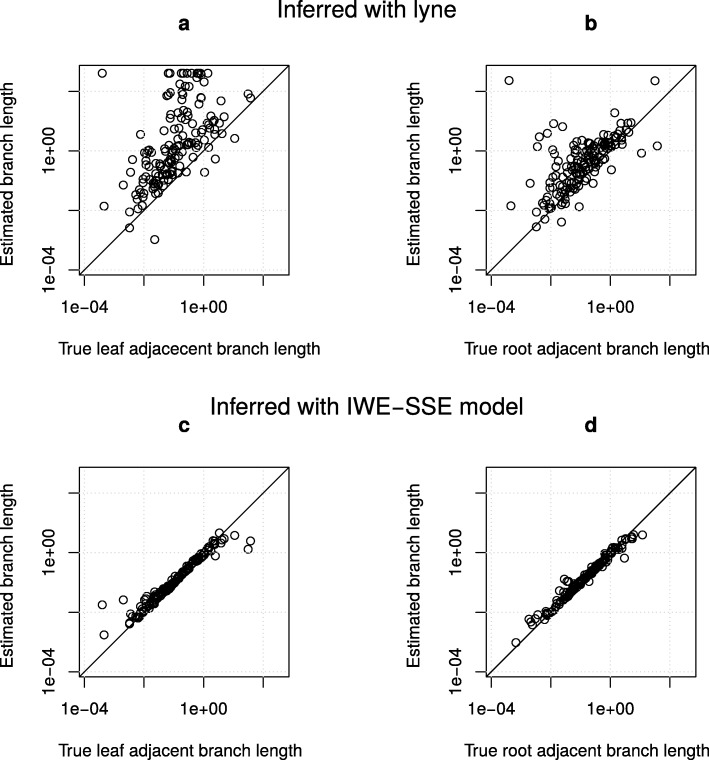
Comparison between inferred branch lengths by lyne (**a** and **b**) and the IWE-SSE inference (**c** and **d**) when data was simulated according to the IWE-SSE model. Plots a and c refer to the branches that are adjacent to the leaves and plots b and d to the branches that are adjacent to the root

When data was simulated according to the model of [4], we observed a slight bias for overestimation in the IWE-SSE inference (online appendix Fig. 6). The lyne estimates showed no obvious bias but were less accurate for longer branches. When data was produced with the IWE-SSE model, lyne substantially over-estimated branch lengths, especially for the leaf-adjacent branches, while IWE-SSE inference was very accurate (Fig. 9).

## Discussion

Our simulation results and the application example with methylation states of haematopoietic cells suggest that the possibility of CpG-island wide methylation changes should be taken into account when analysing methylation dynamics. For the single-site methylation changes (SSEs) we assume in our current model that the new methylation state (unmethylated, partially methylated, or methylated) is independent of the state before the SSE. A possible extension of our model would be to allow for the SSEs and for state-changes within IWEs the class of models proposed by [4], who consider all reversible 3×3 rate matrices for the three states. One could further consider Ising model based constraints on regions or islands [17], since the resulting likelihoods can also be calculated in linear time and the number of parameters per region is just three. The efficiency of our method, however, relies on the conditional independence of CpG sites given the IWE state of the CpG island because this allows us to carry out MCMC steps only for entire CpG islands and calculate marginal likelihoods for the CpG sites with an efficient dynamic programming approach. Allowing that SSEs depend additionally on the current states of neighboring CpG sites would require separate MCMC steps for all CpG sites. This would substantially slow down our MCMC method, especially if more than just the two directly neighbored CpGs have to be evaluated in each of these MCMC steps [21, 22, 24].

Even though we assumed a priori a constant IWE rate in our model, we obtained clear evidence that the number of IWEs per branch length unit (which summarizes expected numbers of IWEs and SSEs) varies among the branches (Fig. 6). Further, our results suggest that overall methylation frequencies vary among the branches (Fig. 3). Also this is not explicitly taken into account in our model, as we assume that IWEs have their probabilities sampled from the same Dirichlet distribution across the tree. However, compound Poisson based models [15] of genome wide change are natural extensions to our framework. Thus, we could allow for genome-wide events that modify the IWE rate and the parameters of the Dirichlet distribution from which the methylation state distribution are sampled in IWEs. An alternative approach, in analogy to some relaxed molecular-clock models in phylogenetics [6], would be to assume that IWE rates or other parameters are sampled from a prior distribution independently for each branch.

Further comparisons of the performance of the IWE-SSE model on data procured in vitro experiments of germ cell differentiation suggested that inference with the IWE-SSE model is more accurate than inference without IWEs or inference with the lyne software package, as estimates were closer to a linear model of ground truth time spans. However all inferences produced a deviation from proportionality, by overestimating branch lengths in the early stages of the experiment when cells were in the EpiLC stage.

We went on to further estimate branch lengths in this datasets using the Hamming distance and an euclidean mean based distance. Both were outperformed by the IWE-SSE inference tool.

In the application example with the data of [2], the tree topology and the methylation states at the internal nodes were given. Our computational approach for the IWE-SSE model can also be adapted to reconstruct genealogies when methylation states are given only for the tips of the tree and combined with methods to explore possible tree topologies [10]. A potential application area could then be the inference of genealogies of cells sampled from neoplasms, e.g. to reconstruct the growth and mutation history of cancer clones [3, 31]. Accounting for IWEs may not only improve the accuracy of inferred cell genealogies but also allow for a better detection of aberrant methylations, which are a known hallmark of cancer [12]. The best possible data for reconstructing cell genealogies from methylation patterns would obviously be single-cell methylation data. To our knowledge, however, it is not yet possible to generate such data. Therefore the possibility of inferring single cell genealogies from long-read methylation data, which are now becoming available [27], constitutes a promising avenue of research.

## Conclusion

We found that the model with CpG-island wide methylation rate changes (IWEs) fit the methylation data from murine haematopoietic cells significantly better than a model without IWEs. Furthermore, the IWE-SSE model detected certain developmental phases in haematopoiesis that many CpG islands were affected by IWEs, which may indicate enhanced activity in gene regulation. Our simulations comparing the inference methods based on the IWE-SSE model to inference method that assume independence between CpG sites show the necessity of modeling simultaneous changes of methylation states in CpG islands in addition to single-site changes. This view is further affirmed by comparison between ground truth times in cultured cells with estimated branch lengths. The IWE-SSE model produces a better proportional fit than do models not accounting for IWEs.

## Supplementary information

**Additional file 1** Online appendix.

## Abbreviations

Eryth: nucleated erythrocyte
hEpiLCs: human epiblast-like cells
hPGCs: human primordial germ cells
HSC: haematopoietic stem cells
IWE: island-wide events (changing methylation frequencies in a CpG island)
MCMC: Markov Chain Monte Carlo
MEP: megakaryocyte-erythroid progenitor cells
MPP1/2: multipotent progenitors 1/2
SSE: single-site events (changing methylation at a single CpC site)

## Footnotes

1 https://www.kostkalab.net/software.html
